# A Narrative Review on Oral and Periodontal Bacteria Microbiota Photobiomodulation, through Visible and Near-Infrared Light: From the Origins to Modern Therapies

**DOI:** 10.3390/ijms23031372

**Published:** 2022-01-25

**Authors:** Andrea Amaroli, Silvia Ravera, Angelina Zekiy, Stefano Benedicenti, Claudio Pasquale

**Affiliations:** 1 Department of Orthopedic Dentistry, Faculty of Dentistry, I.M. Sechenov First Moscow State Medical University, 119991 Moscow, Russia; zekiy82@bk.ru; 2 Department of Surgical and Diagnostic Sciences, University of Genoa, 16132 Genoa, Italy; stefano.benedicenti@unige.it (S.B.); clodent@gmail.com (C.P.); 3 Department of Experimental Medicine, University of Genoa, 16132 Genoa, Italy; silvia.ravera@unige.it

**Keywords:** gum disease, laser therapy, light therapy, low-level laser therapy, microbiome, mucositis, periodontitis, prokaryote, periodontal disease, oral infection

## Abstract

Photobiomodulation (PBM) consists of a photon energy transfer to the cell, employing non-ionizing light sources belonging to the visible and infrared spectrum. PBM acts on some intrinsic properties of molecules, energizing them through specific light wavelengths. During the evolution of life, semiconducting minerals were energized by sun radiation. The molecules that followed became photoacceptors and were expressed into the first proto-cells and prokaryote membranes. Afterward, the components of the mitochondria electron transport chain influenced the eukaryotic cell physiology. Therefore, although many organisms have not utilized light as an energy source, many of the molecules involved in their physiology have retained their primordial photoacceptive properties. Thus, in this review, we discuss how PBM can affect the oral microbiota through photo-energization and the non-thermal effect of light on photoacceptors (i.e., cytochromes, flavins, and iron-proteins). Sometimes, the interaction of photons with pigments of an endogenous nature is followed by thermal or photodynamic-like effects. However, the preliminary data do not allow determining reliable therapies but stress the need for further knowledge on light-bacteria interactions and microbiota management in the health and illness of patients through PBM.

## 1. Light Is Life: The Origin

Life on Earth arose through a lengthy abiogenic process that began with the prebiotic synthesis of elementary organic compounds on its surface and in the atmosphere during the Hadean eon [1]. Throughout Darwinian evolution, reproduction, mutation, and natural selection were crucial events, and the non-biological processes involved in the formation of organic matter followed a chemical evolution of increasing complexity, which required molecular self-replication, self-assembly, and autocatalysis [1,2]. The conversion of physical energy, such as irradiated sunlight, geothermal events, and lightning high-energy electrical discharges, in mechanical work probably led to organizational changes of molecules and polymers from pre-cell structures to living cell systems [3]. The primitive Earth had higher energy solar radiation compared with today because of the young solar-type star features and the different atmospheric composition, such as the absence of an ozone layer [4]. The photochemistry benefited from these primordial conditions.

Different models of prebiotic Earth were proposed by Oparin [5], Urey [6], and Rubey [7]. Subsequently, in 1962, Holland [8] suggested a multi-stage model for early atmospheric evolution to reconcile the two previous contrasting hypotheses. However, the debate has continued to evolve over time. Regardless of the different hypotheses, the prebiotic synthesis of organic matter in the atmosphere or the air-ocean interface, as well as its possible origin from meteoritic and cometary debris, clearly constituted the spark of life.

The physicochemical conditions of the primitive Earth supported the chemical reactions that generated simple organic compounds from inorganic precursors. These water-soluble organic molecules underwent subsequent reactions to generate structures with increasing complexity and new properties [9,10]. In this regard, Baur [11] showed that the spontaneous formation of amino acids is thermodynamically possible in systems containing carbon dioxide, nitrogen, iron(II)-containing minerals, and water, with solar radiation constituting the energy source. Indeed, the distinctive electronic structure of copper, iron-sulfur, and manganese oxides in the soils and sediments can be excited by absorption of photo-energy, allowing conversion of the solar energy to electron energy and photoelectron reduction [12].

In the evolution of photocatalysis of primordial molecules, the abiogenic origin of proteinoids, flavins, and pteridines has played a pivotal role because they represent the most ancient components of the metabolic system [13] and may be correlated with the primordial cell origin. Novel model systems have demonstrated that these molecules exhibit the physiological reception of light, and researchers have proposed their ability to spontaneously aggregate in aqueous media and organize microspheres [13,14,15,16]. Bahn and Fox [17] were the first to show that proteinoid microspheres can mediate the photocatalytic phosphorylation of adenosine diphosphate (ADP) into adenosine triphosphate (ATP). Therefore, flavins and pteridines could exert a key role in the light energy transformation into the macroergic bonds of ATP [18].

In addition, models of abiogenic synthesis by Krasnovsky and Umrikhina [19] and Szutka [20] provide evidence for the presence of the pigment protoporphyrin IX on primitive Earth. Because protoporphyrin IX is fundamental in biosynthetic pathways of the chlorophyll and cytochrome heme group [21], researchers hypothesized its contribution to the formation of the reaction centers as photo-assimilators. Indeed, Lozovaia et al. [22] showed a change in the absorption spectrum and a photochemical activity increase in the pigment due to the embedding of a low-molecular-weight amino acid polymer as proteinoids. Kolesnikov et al. [23] studied the activity of the photo-phosphorylating system in suspensions, showing that cytochromes can act as primitive and alternative electron acceptors. The results suggest that in those conditions, the photochemical process of ATP formation could be linked to the functioning of free-radical flavin molecules [24]. In addition, researchers suggest a role in the first peptide-nucleic acid formation [23,24,25]. In other words, life needed adequate energy to exist, and the emergence of a self-contained chemical system was critical in order to support a primitive metabolism, the generation of information-containing molecules and their ability to be copied, and for the self-assembly of enclosed membranes [26], paving the way to various scenarios of evolution by selection.

## 2. Light Is Life: The Evolution

Metabolism of the first proto-cells was influenced by the primordial atmosphere composition, characterized by low oxygen levels and abundance of carbon dioxide and methane. In this condition, hydrogen, hydrogen sulfide, and methane represented the electron donors, while the proto-cell could not dissociate water due to a low-energy state [27]. This scenario was maintained for a billion years up to the evolution of primitive photosynthesis by proto-bacteria—the cyanobacteria—3500 million years ago. The increased oxygen in the atmosphere drastically modified Earth’s environment up to the Great Oxidation Event, characterized by an enormous mass extinction of Earth’s primitive anaerobic life forms, caused by the accumulation of lethal oxygen concentrations in Earth’s atmosphere [28]. In other words, proto-bacteria were better able to cope with the ‘evolutionary challenge’ of oxygen, compared with the anoxic bacteria, which survived only in environmental niches where no oxygen could penetrate. Indeed, aerobic metabolism provided the proto-bacteria with a high energy boost that facilitated evolution [27,29].

From an evolutionary point of view, a review of the current state of the art by Degli Esposti [30] showed that members of the heme-copper-oxygen family evolved from iron-oxidizing proteobacteria, resulting in an improved reduction of oxygen to water. Researchers have proposed that evolution occurred through nitric oxide (NO) reductases containing an iron atom instead of a copper atom in the catalytic center; these enzymes could thus reduce NO instead of oxygen [30,31]. However, NO reductase evolution seems to be more recent than the heme-copper-oxygen family. Hence, it has been assumed that the acidophilic iron (II)-oxidizer groups observed in ancestral bacteria related to *Acidithiobacillus* might have been involved as the primordial form in the evolution of the haem-copper-oxygen family and cytochrome c oxidase (COX), which contains two heme groups and two copper centers [30]. The formation of an efficient molecular apparatus to produce metabolic energy occurred early in evolution [13]. Indeed, the archaeal, bacterial, and eukaryotic electron transport systems have essentially the same degree of complexity [31]. However, bacterial electron transport chains (ETC) are usually shorter and possess lower phosphate/oxygen ratios than the mitochondrial transport chain [31].

The origin of respiratory terminal oxidases predated the evolutionary split between bacteria and archaea, and the auxiliary proteins catalyzing the supply of reducing equivalents evolved in parallel [31,32,33]. Complexes between cytochrome b and Rieske iron-sulfur proteins (with 2Fe–2S being the simplest cluster) probably appeared very early as ancestral complexes, thanks to their ability to work as an energy converter under anaerobic conditions [31]. Thus, because early evolution involved adaptation to environmental changes and the ability to manage metabolic energy, the historical origin of respiration and energy-conserving ATP synthases is similar for all life forms [31,34].

Generally, prokaryotic cells possess an ETC composed of several enzymatic complexes embedded in the plasma membrane that, in aerobic conditions, use reduced co-enzymes such as NADH as electron donors. Specifically, the electron transfer occurs through the iron-sulfur proteins and the cytochromes containing heme groups and copper, magnesium, and zinc ions to the final acceptor, usually oxygen. A proton gradient is simultaneously accumulated, which is necessary for ATP synthesis through the FoF1 ATP synthase [35,36]. However, adaptation to environmental conditions has led to an enormous variety of bacterial ETC that, in part, can support its evolutionary history from the first ancestral aerobic bacterium to the mitochondria of eukaryotes [37,38]. For example, *Paracoccus denitrificans* is a facultative anaerobic prokaryotic model that, in aerobic conditions, principally synthesizes ATP using the oxygen as the terminal electron, through the ETC formed by four complexes, like those expressed in the mitochondrial inner membrane [37,38]. In addition, *P. denitrificans* can adopt the aerobic metabolism according to oxygen limitations by expressing an oxidase with a relatively low or high affinity for oxygen [39]. However, when *P. denitrificans* grows anaerobically, nitrate replaces oxygen as the electron acceptor, the ETC is structured differently, and oxidative phosphorylation proceeds via anaerobic respiration [39,40]. In this case, the first reduction step is catalyzed by nitrate reductase consisting of three different subunits with heme groups, iron-sulfur centers, and a molybdenum cofactor. Subsequently, the process is carried out by: (i) a nitrite reductase homodimer containing a heme group; (ii) nitric oxide reductase consisting of two subunits with heme C, heme B, and non-heme iron; (iii) nitrous oxide reductase, a homodimer that contains two copper centers, which generate dinitrogen; and (iv) denitrification. Through nitrate respiration, the membrane-bound nitrate reductase induces a hydrogen ion concentration gradient by which ATP synthase catalyzes ATP synthesis [39,40,41,42]. The anaerobic formation of ATP can, however, occur by incorporating the respiratory ETC and using other terminal electron acceptors instead of nitrogen, such as sulfate or sulfur ions. Because these molecules have a lower reduction potential than oxygen, less energy is formed in anaerobic versus aerobic conditions [42].

It is clear that the evolution of increasingly complex cells and organisms like the origin of the building blocks of life are based on debated enigma and theory, which are hardly proven. For example, the origin of eukaryotes and their mitochondria has been presented in the literature through more than twenty different versions of endosymbiotic theories [43]. In addition, non-endosymbiotic theories have been formulated, despite molecular evolutionary studies more or less disproving non-symbiotic models for the origin of plastids and mitochondria [43,44]. On the other hand, as Martin et al. [44] concluded: “early archaeal evolution and the origin of eukaryotes are ancient events, so ancient that they push phylogenetic methods to their limits, and possibly beyond”, probably because many of those events were played by ‘actors’ that went extinct.

The most accredited version of the endosymbiotic theory postulates that mitochondria and plastids were once free-living prokaryotes and became organelles of eukaryotic cells [45]. The last eukaryotic common ancestor (LECA) probably displayed the first symbiosis with a facultative anaerobic alpha-proteobacterium to originate the ‘universal’, non-obligatory anaerobic mitochondrion. Moreover, mitosomes and hydrogenosomes evolved from mitochondria based on the ecological niche colonized by the host [45,46], suggesting a common origin. However, was the LECA a prokaryote (H_2_-dependent methanogenic archaeon) or a eukaryote without mitochondria (proto-eukaryote)? The first scenario seems more plausible, and eukaryotic cellular complexity (nucleus and mitochondria) would have arisen after endosymbiosis [43,44,45,46,47,48]. The symbiosis between an ancestral facultative anaerobic eukaryote and a cyanobacterium has also led to three plastid lineages: *Glaucophytes*, *Chloroplastida*, and *Rhodophytes* [45].

The detailed characterization of bioenergetic organelles of eukaryotic cells has contributed to affirming their bacterial origin. Indeed, their ETCs resemble those of free-living bacteria, but have been tailored through reductive and expansive events according to the host cell [47]. In addition, ontogenetic cycles and multicellular organization in eukaryotes caused further bioenergetic organelle modifications [47,48]. On the other hand, eukaryotes show other ETCs, such as the plasma membrane redox and cytochrome P450 (CYP) systems, which have simpler organization and non-bioenergetic functions [47,49,50].

## 3. Light Is Life: The Therapy

### 3.1. Photobiomodulation

Photobiomodulation (PBM), previously known as Low-Level Laser Therapy (LLLT), causes cell manipulation by a photon energy transfer employing non-ionizing light sources in the visible and infrared spectrum, including lasers, light-emitting diodes (LEDs), and broadband light [51]. This therapy, based on non-ablative energies, is a non-thermal process, which involves endogenous photo-acceptors eliciting photophysical (i.e., linear and nonlinear) and photochemical events at various biological scales [51].

**Figure 1:**
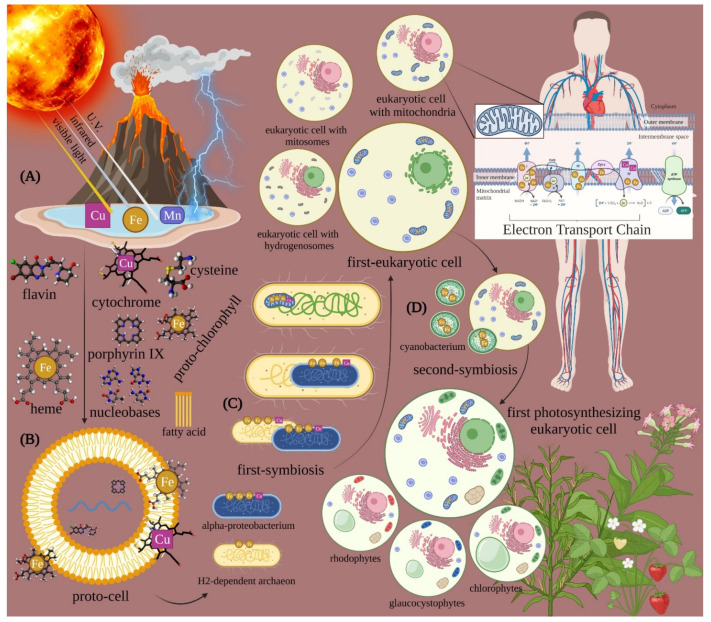
Parallel origin and evolution of life and photoacceptors. The conversion of physical energy (sunlight, geothermal events, lightning) in mechanical work led to the organization of complex molecules and polymers (**A**). Copper (Cu), iron (Fe), and manganese (Mn) could have been excited by the absorption of photon energy. This event allowed the generation of structures with increasing complexity, which after incorporation of those minerals, worked as ancient components of the metabolic system. These primordial cytochromes, porphyrins, chlorophylls, pigments, flavins, pteridines inherited the ability to interact with light and spontaneously aggregated through fatty acid in microspheres (**B**). Peptide-nucleic acid formation also occurred. A first proto-cell formed, which was able to produce energy (ATP) through the photocatalytic phosphorylation of ADP and make copies of itself thanks to the generation of information-containing molecules. Eukaryotic cells arose through a first-symbiosis between an H2-dependent methanogenic archaeon and a facultative anaerobic alpha-proteobacterium, which became the “universal” non-obligatory anaerobic mitochondrion and contributed to the nucleus formation (**C**). Moreover, mitosomes and hydrogenosomes evolved from this mitochondrion based on the ecological niche colonized by the host. A second symbiosis between the facultative anaerobic first-eukaryotic cell and a cyanobacterium (**D**) led to an ancestral plant cell, which was followed by three plastid lineages: chloroplastida, glaucophytes, and rhodophytes. Therefore, metals and molecules that are able to be energized by photons have been transmitted through evolution from the life origin into the primordial broth to prokaryotic and eukaryotic cells, where are involved in their metabolism and physiology.

Although humans and animals have not utilized light as their primary energy source, many molecules involved in their physiology have retained their primordial photoacceptive properties, inserting the energized molecules into the first proto-cells and then into prokaryotes outer membrane during their evolution (Figure 1). Afterward, these molecules were inserted into the inner membrane of the mitochondria in free eukaryotic cells, thus becoming part of their physiology. In many cases, the energized molecules gradually lost the possibility to utilize direct light interaction. However, photoacceptors can be modulated by PBM at specific wavelengths of light, thus influencing cell physiology.

The PBM primary mechanisms are based on the Grotthuss–Draper law (principle of photochemical activation), which states that only the light absorbed by a system can bring about a photochemical change [52].

Pastore et al. [53] showed that COX (respiratory complex IV) acts as a photoacceptor at 632.8 nm due to two heme A moieties and two copper centers. On the other hand, COX also displays absorption peaks at 450, 620–680, and 760–895 nm [54]. Moreover, we have shown that 808 and 980 nm selectively stimulated complex IV and, in part, complex III, which contains a cytochrome b subunit with two heme moieties, a cytochrome c1 subunit with one heme group, and a Rieske protein subunit (UQCRFS1) with a 2Fe-2S cluster [55,56]. Conversely, 1064 nm wavelength affect complex I (with eight 2Fe-2S clusters), in addition to complexes III and IV [57]. The extrinsic mitochondrial membrane complex II (with a heme B prosthetic group) does not seem receptive to photons at these wavelengths [54,55,56,57].

The visible 400–500 nm wavelengths excite flavins and flavoproteins [58]. Thus, light could act on different cell pigments and respiratory complexes I and II [59]. In addition, porphyrins, heterocyclic organic compounds complexed to hemoglobin, CYP enzymes, and complex IV possess the ability to absorb light at 400–420 nm [60] and 450 nm [61]. Heme-containing protein and di-nitrosyl iron complexes form complexes with NO (i.e., NO-hemoglobin) as well as the thiol groups (i.e., S-nitrosothiols), and light may induce NO release from a variety of cellular sources [62]. Lastly, near-infrared light seems to excite water, affecting temperature-gated calcium (Ca^2+^) ion channels [63] and lipids that show a mild but significant absorption peak in the range of 900–1000 nm [64]. Meanwhile, visible light modulates the structure and activities of the opsin proteins family, which are involved in cellular pathways of different cell types [65].

The primary PBM targets are linked to the endogenous release of reactive oxygen species (ROS) and NO, ATP production, and modulation of Ca^2+^ fluxes and redox homeostasis, which can play a key role in cell proliferation, growth, and apoptosis [66,67,68]. Therefore, PBM therapy seems to support treatments in many medical and veterinary areas to restore cell dysfunction and promote recovery from illness [62,69,70,71,72,73,74,75]. PBM therapy has been recommended unequivocally for oral mucositis prevention in patients treated with chemotherapy by the Mucositis Study Group of the Multinational Association of Supportive Care in Cancer/International Society for Oral Oncology [76]. However, concerns have been raised regarding the potential stimulatory effect on existing malignant or pre-malignant cells and induction of therapeutic resistance [77,78,79]. Chemotherapy also supports the progression of gingivitis and periodontitis by bacterial pathogen growth [80]. Therefore, because of the ubiquitous presence of the primary targets of PBM in all kingdoms of life [70], the modulatory effect of light therapy on the prokaryotic communities needs attention in view of the role of microbiota in human health. In that regard, Liebert and colleagues recently introduced the term ‘photobiomics’ to represent the PBM effects on microorganisms [81].

### 3.2. The Oral Microbiota in Health and Disease

The neo-Darwinian evolution has worked to distance and increase the complexity among the protocell, bacteria, and mammals. However, bacteria and humans have organized a coevolutionary and mutualistic relationship for billions of years. Therefore, a modern vision in medicine considers the human body as a complex assemblage of eukaryotic and prokaryotic cells organized into functional organs, tissues, and cellular communities. Ninety percent of the cells in and on the human body are microbial cells and, despite the presence of viruses, archaea, yeast, and protozoa, the most represented community is bacteria. In the human body, the entire microbial community, called microbiota, is principally formed by four phyla—*Actinobacteria*, *Firmicutes*, *Proteobacteria*, and *Bacteroidetes*—which colonize the oral cavity, esophagus, skin, vagina, and gut [82]. Microbiota colonization occurs and is modified quickly in the early years of life, while in an adult, it remains relatively stable and is unique to each person [83]. However, the microbiota is a living ecosystem undergoing growth rate fluctuations and survival because of changes in diet, vigorous cleaning and disinfection, lifestyle, drugs (i.e., antibiotics), and diseases [82,83]. The constitutional microbiota may re-emerge when the original conditions resume [82,83]. The human microbiota consists of a core part, relatively constant in all the individuals, and a variable part associated with the individual case history. During its life cycle, the microorganisms belonging to the microbiota interact with each other and the human host cells through intraspecies or interspecies communication. Bacteria can modulate tissue signaling pathways and immune cell responses. Moreover, they produce vitamins (i.e., cobalamin) and bacteriocins, molecules able to inhibit or kill bacteria. In other words, the microbiota causes beneficial or detrimental changes in the host [84]. Indeed, dysbiosis—loss of balance within a human-associated microbial community—is associated with several pathological conditions, such as insulin resistance in patients with type 2 diabetes, esophagitis and Barrett’s esophagus, ulcers, inflammatory bowel disorder (Crohn’s disease), recurrent abdominal pain, vaginitis, arthritis, autism, neurodegenerative diseases, cancers, collateral periodontitis, and macular degeneration [82,85,86,87]. Microbiome characterization offers an opportunity for innovative diagnostic biomarkers and therapy.

The oral cavity harbors over 700 species of bacteria and represent the second-largest heterogeneous microbiota of the human body, after the gut [88]. Bacteria can colonize two different surfaces in the oral cavity: the hard tissue of the teeth and the soft tissues of the oral mucosa of the tongue, cheeks, gingival sulcus, tonsils, and palate as well as saliva. Deo and Deshmukh [88] showed that the principal bacterial genera found in the healthy oral cavity are:

- gram-positive: cocci—*Abiotrophia*, *Peptostreptococcus*, *Streptococcus*, and *Stomatococcus*; rods—*Actinomyces*, *Bifidobacterium*, *Corynebacterium*, *Eubacterium*, *Lactobacillus*, *Propionibacterium*, *Pseudoramibacter*, and *Rothia*;
- gram-negative: cocci—*Moraxella*, *Neisseria*, and *Veillonella*; rods—*Campylobacter*, *Capnocytophaga*, *Desulfobacter*, *Desulfovibrio*, *Eikenella*, *Fusobacterium*, *Hemophilus*, *Leptotrichia*, *Prevotella*, *Selemonas*, *Simonsiella*, *Treponema*, and *Wolinella*;
- the uncultured divisions GN02, SR1, and TM7 [89].

For further information, please consult the Human Oral Microbiome database website www.homd.org (30 December 2021) and the NIH Human Microbiome Project https://www.hmpdacc.org/ (30 December 2021). This commensal microbiota plays a key role in maintaining oral and systemic health [90] through bacteriocin and biofilm formation against pathogens (colonization resistance).

Dysbiosis paves the way for opportunistic pathogens such as *Candida* spp. and *Staphylococcus* spp. [88]. In addition, this condition exhibits cariogenic properties by *Streptococcus mutans*, *Actinomyces naeslundii*, *Propionibacterium* spp., and *Lactobacillus* spp., or to induce periodontitis and halitosis by *Streptococcus salivarius*. Periodontitis is also favored by the colonization of the periodontal pocket and its infection with *Porphyromonas gingivalis*, *Treponema denticola*, *Anaeroglobus geminatus*, *Tannerella forsythia*, *Filifactor alocis*, *Eubacterium saphenum*, *Prevotella denticola*, *Prevotella intermedia,* and *Porphyromonas endodontalis* [89,90,91]. *Escherichia coli*, *Pseudomonas aeruginosa, Enterococcus faecalis*, and *Staphylococcus aureus* were found to colonize the oral cavity of hospitalized patients, and the presence of *Helicobacter pylori* in dental plaques was directly associated with gastric infection [90]. Dentine lesions facilitate anaerobic proteolytic bacteria and enterococci. Lastly, *P. gingivalis* and *Fusobacterium nucleatum* may provoke oro-digestive cancers and oral squamous cell carcinoma [90,92]. Oral infection can gain access to the bloodstream and cause infectious endocarditis; brain, kidney, and liver abscesses; rheumatoid arthritis; Alzheimer’s disease and dementia; as well as pregnancy-related complications [89,91]. As a result, the human microbiota is an emerging target for the development of a modern therapeutic approach to several human diseases.

### 3.3. Photobiomodulation on Bacterial Microbiota

#### 3.3.1. Evidence-Based Literature

In the past, most infections of odontogenic origin have been managed by dentists through antibiotics therapy and prophylaxis [93]. However, the ability of bacteria to survive in drug concentrations that should kill or inhibit them, and their routinely indiscriminate prescription, has allowed antibiotic resistance to occur [94,95]. Despite, in some cases, the prescription of antibiotics being essential, the risk of antibiotic toxicity and allergies can limit their applicability. Thus, the light could be a suitable alternative cure supporting oral infection prevention and cure. Indeed, in nature, solar radiation is shown to select for pigmented bacteria [96]. Culture-to-culture physical interactions mediated by biophoton visible and near-infrared light emission were also preliminarily described in *E. coli* cultures [97]. On the other hand, UV irradiation is well-known to photo-destroy bacteria. Unfortunately, even minimal overexposure to UV is dangerous to healthy tissue [98]. Additionally, applying local or systemic exogenous photosensitizers, inappropriate cells can be destroyed by specific light wavelengths. This application is known as photodynamic therapy [99].

We previously discussed how PBM, through the interaction of visible and near-infrared light with endogenous photoacceptors, can positively affect normal eukaryotic cell metabolism and support recovery from disease. Therefore, according to the PBM mechanism of action and the prevalence of molecular photoacceptors in all life forms, the PBM therapy could also affect bacteria cells.

The literature about bacteria and photobiomodulation discussed herein was screened through keywords such as bacteria, microbiota, microbiome, low-level laser therapy, light therapy, and photobiomodulation on PubMed and Scholar databases. Articles were also selected from the references of papers reviewed.

Bicknell et al. [100] showed that PBM at 660 and 808 nm influenced the gut microbiota of mice. Infrared light particularly affected *Allobaculum* cells, which increased their growth. Using the same wavelength, Thomé Lima and collaborators suggested that PBM can improve mouse wound healing by killing or inhibiting *Pantoea agglomerans* bacterium [101]. Similarly, faster healing and regeneration were observed by Amaroli and colleagues in *Dendrobaena veneta* after irradiation with 808 nm PBM, where the therapy significantly decreased bacterial load [102].

The PBM also seems to influence the bacteria cell cycle that regularly or occasionally forms the oral microbiota in healthy and/or ill patients (Table 1). Indeed, literature shows that *P. gingivalis, F. nucleatum, S. mutans,* and *E. faecalis* exposed to visible light at wavelengths of 400–500 nm, at power densities between 0.26 and 1.14 W/cm^2^ (60–180 s), manifested a phototoxic effect [103]. *P. gingivalis* and *F. nucleatum* were more sensible and exhibited effects with the minimal fluences of 16–39 J/cm^2^, while *S. mutans* and *E. faecalis* needed 159–212 J/cm^2^ (Table 2). The effect is not due to an indirect medium modification nor its dangerous increase in temperature. However, an infrared diode laser wavelength of 830 nm did not affect the cells [103]. In the same way, Henry et al. [104], through 488–514 nm laser lights, but lower fluences of 4.2 and 21 J/cm^2^, exerted a drastic phototoxic effect on *P. intermedia.* Only a mild effect was observed on *P. gingivalis*, while *P. denticola* and *P. endodontalis* were not affected.

Because of their feature of black-pigmented bacteria, authors concluded that the nature of the metabolic pathways for porphyrin synthesis could protect *P. denticola* and *P. endodontalis* but made *P. intermedia* more susceptible to damage from these wavelengths. A better effect of the lower wavelengths than that at 800–900 nm was also described by Nussbaum et al. [105] when 0.015 W/cm^2^ and 1–50 J/cm^2^ were irradiated in continuous wave (CW) mode on *P. aeruginosa*, *E. coli*, and *S. aureus*. Specifically, 630 nm appeared most associated with bacterial inhibition compared to 810 and 905 nm. Interestingly, *E. coli* growth was inhibited by 630 nm and 1 J/cm^2^, but significantly increased at 810 nm and 20 J/cm^2^. However, the same team, in a comparative study between CW or frequency-modulated light mode of irradiation of 810 nm (0.015 W/cm^2^; 1–50 J/cm^2^; 26, 292, 1000, or 3800 Hz) showed that laser-mediated growth of *S. aureus* and *E. coli* was dependent on pulse frequency [106]. In addition, *P. aeruginosa* growth increased up to 192%, using 1000–3800 Hz, whereas 26–292 Hz pulsing irradiation produced only a growth trend. All bacteria increased proliferation after irradiation with 810 nm in CW mode.

Different evidence concerning the near-infrared wavelengths and the CW mode of irradiation was described by de Sousa et al. [107] on *S. aureus*, *E. coli*, and *P. aeruginosa* and Dixit et al. [108] on bacterial strains of *P. aeruginosa*, *E. coli*, *E. faecalis*, *S. epidermidis*, *Streptococcus pyogenes*, *Shigella*, *Salmonella* sp., *Staphylococcus saprophyticus*, *Salmonella typhi*, *S. epidermidis*, *S. aureus*, and *Klebsiella pneumoniae.* In detail, *P. aeruginosa* was inhibited at the wavelengths of 660, 830, and 904 at a fluence of 24 J/cm^2^. *E. coli* had similar growth inhibition at a wavelength of 830 nm at fluences of 3, 6, 12, and 24 J/cm^2^. At wavelengths of 660 and 904 nm, growth inhibition was only observed at fluences of 12 J/cm^2^ and 18 J/cm^2^, respectively [107]. Meanwhile, at 810 nm and laser fluences of 13 J/cm^2^, 18 J/cm^2^, and 30 J/cm^2^ had effectiveness in the treatment of Gram-negative and Gram-positive bacteria [108], and the effects were higher in Gram-positive.

De Sousa et al. [109] also reported that 830 and 904 nm wavelengths at a fluence of 3 J/cm^2^ significantly induced topographical changes of the *S. aureus* cell structure. Additionally, *S. aureus, P. aeruginosa*, and *E. coli* growth were inhibited at fluences >6 J/cm^2^ when irradiated with a 450 nm laser light [110].

Near-infrared laser light of 810 nm for 30 s in two cycles with 1.5 W and 1 W exerted an antibacterial effect against three cariogenic bacteria, such as *S. mutans*, *Lactobacillus casei*, and *Actinomyces naeslundii* [111]. *S. mutans* irradiation at 780 nm, 400 mW, 5–20 J/cm^2^, and 250–1000 s [112] decreased the proliferation in a dose-dependent manner.

Plavskii et al. [113] showed that laser radiation of 405 and 445 nm causes growth inhibition in *S. aureus* and *E. coli*. Similarly, blue light wavelengths affected *Prevotella* spp. [114], *P. gingivalis* [115], and *P. aeruginosa* [116], but the effect was more evident in *Prevotella* spp. Based on these data, the blue spectral region radiation, like that previously shown with cyan light, may act through a sensitizing effect of endogenous porphyrins and flavin-type capable of inducing reactive oxygen species generation. However, *S. mutans* generally exhibited sensitivity to PBM therapy and was barely affected by blue light when grown in an anaerobiotic environment. Over the range of blue light, 400–410 nm (15 J/cm^2^) but not 430 nm significantly suppressed *P. gingivalis* growth [117]. *S. aureus* cell division has been affected and inhibited by irradiation with 514, 532, and 633 nm [118], while *P. aeruginosa* was stimulated.

The suggestions derived by the Karu experiments indicate support of *E. coli* cell proliferation after irradiation with a wide range of wavelengths and doses [119,120,121]. Furthermore, Bertoloni et al. [122] described the stimulatory effect of 632.8 nm of light (4 J/cm^2^) on *E. coli*, since the cells exhibited enhanced cell metabolism and intensified synthesis of cytoplasmic membrane proteins, increased cell volume, and ribosomal content. Dadras et al. [118], in contrast to its results on *S. aureus*, observed that *P. aeruginosa* increased the cell multiplication when exposed to PBM at 514, 532, and 633 nm.

Lastly, in vivo experiments on rats [123] affected by periodontitis induced by 5-fluorouracil chemotherapy describe the positive effect of scaling and root planing associated with multiple PBM sessions (660 nm; 0.035 W; 4.2 J; 120 s) on periodontitis recovery. The effect involved the response to the therapy of the oral microbiota such as *Aggregatibacter actinomycetemcomitans*, *P. gingivalis*, *Prevotella nigrescens*, and *F. nucleatum*.

The absence of comparative and exhaustive studies does not allow extrapolating reliable clinical approaches but only therapeutic indications. Table 2, however, summarizes the best effects in inducing growth inhibition, cell death, and reduction of biofilm formation. Visible blue and cyan lights seem more effective than near-infrared, probably because of the absorption spectra of porphyrins and flavins to those wavelengths, despite the wide variety of laser parameters employed are not conclusive. However, the data is in accordance with a recent exhaustive review of Leanse et al. [124] showing the antimicrobial effects of blue light (400–470 nm wavelength).

The comparison of research by de Sousa et al. [110] and Nussbaum et al. [105,106] suggest some indications about bacteria-photobiomodulation interactions. *Staphylococcus aureus*, *P. aeruginosa,* and *E. coli* were sensitive to 450 nm irradiation using different intensities, although the effect did not appear strictly correlated to fluences [110], except for *E. coli* that was not affected by 24 J/cm^2^. Conversely, the wavelengths of 630, 660, 810, and 905 nm and a wide range of fluences discordantly impacted the bacteria cell growth [105].

For instance, the 630 nm through 1 J/cm^2^ drastically inhibited *P. aeruginosa* growth, but 2 J/cm^2^ increased it, and the effect took turns at 5, 10, 20, and 50 J/cm^2^. However, at 810 nm, growth increment was observed after irradiation with 1 and 2 J/cm^2^, while the other fluences (5–50 J/cm^2^) inhibited it with different intensities.

In general, the bacteria-photobiomodulation interaction seems not to follow the hormetic behavior of eukaryotic cells, but reflects the windows-effects shown in our previous studies on mitochondria [56].

#### 3.3.2. Possible Mechanism of Action

**Figure 2:**
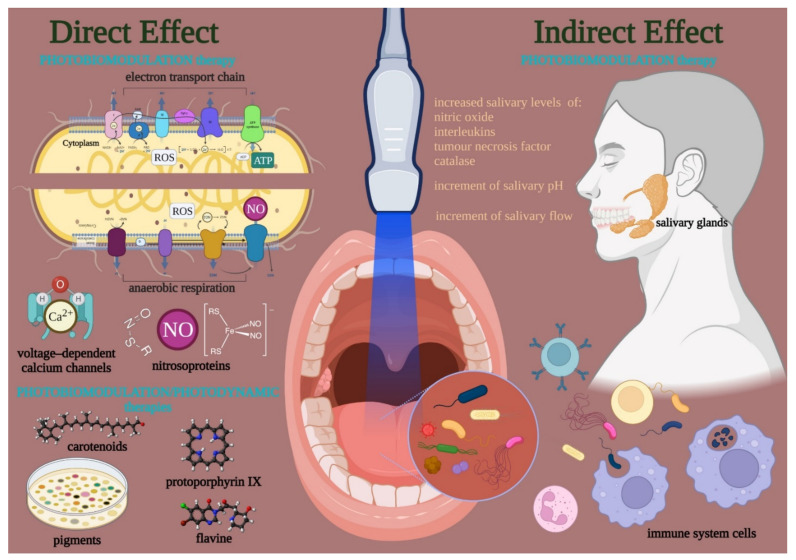
Visible and near-infrared light can modulate the bacteria cell cycle through primary interactions on photoacceptive molecules and pigment targets. A direct effect occurred when the endogenous targets are kept on/in the cell or released in the colony. Conversely, targets in tissues and cells surrounding the bacteria can lead to an indirect effect. The direct effects support a PBM in the strict sense, which modulates the cell metabolism and defense through the photo-energization and the non-thermal effect of light on photoacceptors such as cytochrome, flavins, iron-proteins of the electron transport chain or the anaerobic respiration, nitroso-protein, and voltage-dependent calcium (Ca^2+^) channels; the interaction followed by ATP and reactive oxygen species (ROS) production, nitric oxide (NO) release, and calcium homeostasis modulation. On the other hand, a PBM in a broad sense like a photodynamic effect may occur through the interaction of photons with pigments (i.e., carotenoids, porphyrins) flavins of endogenous nature, which is followed by thermal or oxidative cell damage. In both cases, PBM can modulate the life-and-death mechanisms of the bacteria. However, the PBM may also affect the oral tissue surrounding bacteria and modulate the quality and amount of salivary gland activity and the immune system’s behavior.

Visible and near-infrared light affects the bacteria cell cycle through primary interactions on photoacceptive molecules and pigments (Figure 2). The PBM exerts a direct action when its targets are into the bacterial cell or released in the microorganism colony. However, bacteria can be sensitive to indirect effects exerted by tissues and cells surrounding the bacteria. In the first case, PBM can directly modulate the cell metabolism and defenses through the photo-energization and the non-thermal effect of light on photoacceptors (i.e., cytochromes, flavins, iron-proteins). Conversely, light interaction may also occur through the energization of pigments of endogenous nature, followed by thermal or like-photodynamic effects. In both cases, PBM can determine cell fate [98].

Literature shows that PBM acts on Gram-negative and Gram-positive bacteria without distinction [103,104,105,106,107,108,109,110,111,112,113,114,115,116,117,118,119,120,121,122,123]. Indeed, Dixit et al. [108] described a greater effect of an 810 nm laser irradiation on Gram-positive than Gram-negative. However, the same wavelength similarly affected the two groups when irradiated on *E. coli*, *P. aeruginosa,* and *S. aureus* [104]. Conversely, 400–500 nm prevalently inhibited the cell growth of *P. gingivalis* and *F. nucleatum* (Gram-negative) with respect to *S. mutans* and *E. faecalis* (Gram-positive) [103]. Basically, authors [107] showed that the difference in the PBM therapy efficacy was more correlated with the organism than the group. The wavelengths used in the PBM experiments did not seem to interact with the peptidoglycans or bacterial cell wall lipids. However, these molecules could be sensitive to wavelengths higher than 900 nm, such as 980 nm and 1064 nm [64], since they display some absorption peaks in this spectrum range as well as infrared. The aerobic/anaerobic-facultative or anaerobic metabolism is likewise non-discriminatory and, in both, PBM may occur. Unfortunately, a comparison with strictly aerobic bacteria was not investigated in depth. However, it should be noted that *S. mutans* is sensitive to PBM therapies in a wide range of wavelengths [103,111,112], but the trend changed when the experiments were performed in an anaerobic environment with respect to aerobic growth conditions.

Generally, the effect is correlated with wavelengths, dose, irradiation mode (CW or pulsed), and bacteria strain. For instance, *E. coli* growth can be inhibited or stimulated by different wavelengths, although CW seems the better irradiation mode with respect to pulsed [106,109,110,119,120,121,122]. Visible light at the wavelengths of 514, 532, and 633 nm induced a proliferative effect on *P. aeruginosa* but inhibited *S. aureus*, even if both are aerobic/facultative-anaerobic bacteria [118].

Like in the eukaryotic cell, through the modulation of mitochondria metabolism, bacteria may also be affected by PBM with visible and near-infrared laser light.

Bacterial metabolic conditions, growth phase, and condition of bacterial culture (rich medium or poor medium) seem to affect the PBM effect [125]. Fukui et al. [117] suggested that PBM irradiation might affect *P. gingivalis* metabolism as well as growth, and Basso et al. [112] showed that the induction of cell death in *S. aureus* is mediated by inhibition of its metabolism.

Indeed, the bacteria ETC expresses protein complexes and molecules transferring electrons from an electron donor to an electron acceptor, and, even if the model can be different according to bacterial species, it always leads to ATP production in aerobic conditions [126]. Alongside the water-soluble cytochromes working as electron shuttles, which seem not involved in the light interaction [55], other complexes and cytochromes exhibit macromolecular structures embedded into the cell membrane and show similarity with mitochondria complex I, III, and IV. Mitochondria complex IV has many peaks of absorption from 450 to 900 nm [53] and, along with complex III [55,56,57] and I [57], can be modulated by PBM at 810, 980, 1064 nm of wavelengths. In addition, the succinate-quinone oxidoreductase, an analog of mitochondria complex II [126], could be stimulated by visible light through its flavoproteins. In other words, the PBM can influence ATP production in bacteria, as already observed in eukaryotic cells. However, the ETC is one of the main sites of ROS production in bacterial cells [126,127]. Thus, like in normal and cancerous eukaryotic cells, the different PBM effects on bacterial growth can be correlated to the modulation of energy metabolism and the balance between oxidative stress production and antioxidant defenses. Lushchak reviewed the role of oxidative stress and the mechanisms of protection against it in bacteria [126]. He showed that ROS-induced damage occurs mainly in sites containing iron and copper, causing oxidation of thiol groups of cysteine and methionine, imidazole ring of a histidine, and the rings of tyrosine, phenylalanine, and tryptophan. Additionally, ROS also interacts with DNA and polyunsaturated fatty acids, provoking damage on genome and lipid structures. However, cells display several antioxidant defenses to counteract ROS production. For example, catalase plays a pivotal role in protecting bacteria from oxidative stress, and its iron-containing heme groups can be a target of PBM [127]. We recently showed that 810 nm PBM may reduce the catalase activity in a head and neck squamous carcinoma cellular model, determining an unbalance between oxidative stress production and the antioxidant defenses and stimulating the pro-apoptotic cellular pathways [77].

Conversely, strictly anaerobic bacteria seem to not need respiratory cytochrome oxidases. Nevertheless, functional cytochrome bd-type oxidases with iron groups have been discovered in strictly anaerobic bacteria [128], and heme-proteins play a role in the anaerobic bacterial formation of ATP [37,38,42]. Thus, a similar effect may be assumed on bacteria. Indeed, Lubart et al. [98] reviewed the effect of laser light on bacteria, pointing out ROS production through the effects of PBM on the prokaryote metabolism. They showed that, like in eukaryotes cells, phototoxic effects followed the induction of high amounts of ROS, while low amounts of them promoted proliferation.

Verkhratsky et al. [129] recently reviewed the evolution of Ca^2+^ signaling and Ca^2+^ channels, and they point out that the voltage-dependence of Ca^2+^ channels in *E. coli* resembles that of low-voltage-activated (T) Ca^2+^ channels in eukaryotes. Therefore, like that observed on RBL-2H3 mast cells [130], the PBM could similarly interfere with the bacterial calcium homeostasis and the related processes.

Therefore, bacteria could display primary targets for the interaction with visible and near-infrared light and be affected directly by PBM. However, data are scanty, particularly focused on bacteria responsible for oral cavity disease, and there are no relevant community studies on commensal and pathogens. In addition, the studies are prevalently in vitro and with observational conclusions (growth-stimulating, bacteriostatic, bactericidal effects). Therefore, achieving unequivocal conclusions is nowadays impossible.

Bacteria can also produce endogenous pigments or release them into the biofilm [131,132]. Moreover, in some cases, the association between pigmented and non-pigmented bacteria was observed since, as mentioned above, pigments can generate indirect PBM effects. Bacteria can be killed by light according to the pigment produced and the wavelengths employed [104,114,123]. Specifically, the light energy absorption can generate a thermal increase [133] incompatible with life [98,132], or the energized pigments could increase ROS formation through photodynamic therapy pathways [113]. Therefore, the pigmented bacteria can be killed by light at a low-level dose reliable for PBM, and non-pigmented bacteria associated with the colony can be involved in this lethal effect. This could explain the PBM’s negative effects on *Porphyromonas* spp. and *Prevotells* spp. at the wavelength of 400, 410 nm, or in the range of 400–500 nm, respectively, but not at 810 nm [103,104,117]. On the other hand, both bacteria produce black pigment due to the accumulation of Fe (III) protoporphyrin IX forms, which show the peak of absorption around 400–500 nm [134]. However, differences in endogenous porphyrin structures can modulate the lethality of PBM. For instance, the Prevotellaceae are black-pigmented bacteria accumulating protoporphyrin IX. However, *P. intermedia* integrates that with coproporphyrin III, whereas *P. nigrescens* with uroporphyrin III and heptacarboxyl porphyrin III. This could explain the difference in sensitivity or resistance to PBM observed in these genera.

It is important to note that the indirect effect of PBM should also be considered. For example, saliva plays a pivotal role in maintaining a healthy oral cavity and promotes the natural beneficial relationship between the oral microbiota and the host [135]. The reduced salivary secretion [136], low salivary pH, and altered salivary composition can change the oral cavity microbiota leading to dysbiosis associated with the risk of oral diseases [137]. Photobiomodulation has been employed to improve the functionality of the salivary glands acting on the salivary flow and increasing the salivary pH [138]. Moreover, as shown by Li et al. [139], “PBM therapy increased salivary levels of interleukin-1 receptor antagonist, interleukin-10, total antioxidant capacity, and catalase, and reduced the levels of tumor necrosis factor and interleukin, malondialdehyde, and 8-hydroxydeoxyguanosine”.

Additionally, Ailioaie and Litscher [140] discussed the potential role of PBM in the management of microbiota and the immune system and how the therapy can modulate their interconnection. Therefore, it was proposed that PBM could be beneficial to the normal microbiome recovery, stimulating the immune system [141].

## 4. Light Is Life: Outlook for the Future

Although PBM can affect bacterial cell growth, there are concerns to defining clinical practice guidelines for oral infection and microbiota management since the data shown by the literature are preliminary and prevalently obtained on single pathogen bacteria evaluated in vitro.

Further research on the PBM effect on bacteria metabolism and pigments is necessary to clearly understand the difference among genera and, particularly between commensal and pathogen communities of oral cavities.

Indeed, the possibility to stimulate the production of the bacteriocins and pigments by commensal bacteria and the formation of safe biofilm to improve colonization resistance against pathogens could represent an attractive perspective for new investigations. The peculiar pigments produced by some pathogen or commensal strains could also lead to new strategies of PBM therapy, acting to stimulate eukaryote cell metabolism, tissues healing, and killing bacteria at once. On the other hand, pigments may damage or protect bacteria according to their composition and the wavelengths employed, and the different metabolism exhibited by aerobic and anaerobic bacteria could be a target for therapy able to discriminate and induce opposite effects.

In this respect, a preliminary study by Dai et al. [116] demonstrated that the growth rate inactivation of *P. aeruginosa* cells by blue light was 35-fold higher than that of Human keratinocytes cells. The selective effect on prokaryotic cells with respect to eukaryotic cells by blue light is attributed by Leanse et al. [124] to the high concentration of porphyrins within bacteria relative to mammalian cells; this feature has to be considered to develop effective clinical practices in regenerative medicine. Specifically, to favor the prevention of peri-implant tissue health during radiotherapy treatment and oral disease management, the detection of appropriate clinical photobiomodulation therapies able to stimulate tissue regeneration and counteract or prevent pathogens infection is the challenge in the “photobiomics”. However, more information is necessary to establish the composition and functionality of oral microbiota that arise after pharmacological therapy (antibiotics, chemotherapies), to develop a PBM therapy avoiding the risk to develop harmful biofilms and systemic infections post-treatment. Additionally, the individuation of helpful PBM therapy and probiotic dosing could, together, balance the microbiome and improve the immune system activity of the host to support medical approaches for healthy subjects or patients [140].

Lastly, to develop novel therapies, we should consider the great ability of bacteria to adapt, as exhibited for the drug resistance, which could induce a “light-resistance”, as observed in nature against sunlight [141,142]. Moreover, the PBM effectiveness towards bacterial cells is influenced by the strain of the same bacteria species and its phase of growth, exponential vs. stationary phase [104,122].

## 5. Conclusions

The visible and near-infrared wavelengths can affect bacterial growth. Limits in number and perspective of the literature do not allow tracking an unequivocal mode of action. However, the history of life on Earth supports the mechanism of light-cell interaction. Specifically, the first semiconducting minerals energized through the Sun-radiation on primitive Earth have become the molecules able to absorb photon-energy belonging to the ETC expressed into the membrane of the prokaryotes and mitochondria, influencing the eukaryotic cell physiology. Therefore, also in bacteria, PBM can affect cellular metabolism, homeostasis, defense to stress, and life-and-death mechanisms. Preliminary data do not allow determining reliable therapies, but stress the needing for further knowledge on light-bacteria interaction and microbiota management in health and illness patients through PBM.

## Figures and Tables

**Table 1:** Literature evidence of the photobiomodulation effects on bacteria, employed experimental parameters, and conclusions.

Reference	Bacteria	Parameters	Authors Conclusions
[103]	*Porphyromonas gingivalis*, *Fusobacteriurn nucleatun*, *Streptococcus mutans*, *Streptococcus (Enterococcus) faecalis*	Halogen lamps (400–500 nm), 0.260–0.416 W/cm^2^, 16–75 J/cm^2^, 1 cm^2^, 60–90–120–150–180 s. Plasma-arc (450–490 nm), 1.144 W/cm^2^, 69–206 J/cm^2^, 1 cm^2^, 60–90–120–150–180 s, CW. LED (450–480 nm), 0.520 W/cm^2^, 31–94 J/cm^2^, 1 cm^2^, 60–90–120–150–180 s, CW Diode laser (830 nm), dose described above	Visible light sources without exogenous photosensitizers have a phototoxic effect mainly on Gram-negative periodontal pathogens. 830-nm did not affect the bacteria.
[104]	*Porphyromonas endodontalis*, *P. gingivalis*, *Prevotella intermedia*, *Prevotella denticola*	Argon laser (488 nm and 514 nm), 0.58 W, 20–200 J/cm^2^, 3.5–4 cm^2^, 120–1380 s, CW	Protoporphyrin IX content in black-pigmented bacteria is not the principal factor determining photosensitivity. Oxygen is required during irradiation for black-pigmented bacteria species to be affected. Non-black-pigmented bacteria are much less sensitive to irradiation than black-pigmented bacteria.
[105]	*Pseudomonas aeruginosa*, *Escherichia coli*, *Staphylococcus aureus*	Argon-ion pumped tunable dye laser (630–660 nm); Diode lasers (810–905 nm), 0.015 W/cm^2^, 1–2–5–10–20–50 J/cm^2^, 66–132–330–658–1320–3300 s	Photobiomodulation applied to wounds in the range of 1–20 J/cm^2^ may produce changes in bacterial growth of considerable importance for wound healing. A wavelength of 630 nm is most commonly associated with bacterial inhibition.
[106]	*S. aureus*, *E. coli*, *P. aeruginosa*	Diode laser (810 nm), 0.015 W/cm^2^; 1–50 J/cm^2^; 66–3290 s, CW or 50% duty cycle; 26–5000 Hz	Modulation frequency and radiant exposure of 810 nm laser irradiation significantly influence the effect on particular bacteria. Pulsed laser, at least at a wavelength of 810 nm and high pulse frequency, seems to have the potential to induce growth effects in *P. aeruginosa.*
[107]	*S. aureus*, *E. coli*, *P. aeruginosa*	Diode laser (660, 830 nm), 0.03 W, 3–6–12–18–24 J/cm^2^, 100–200–400–600–800 s, CW Diode laser (904 nm), 0.04 W, 3–6–12–18–24 J/cm^2^, 75–150–300–450–600 s	Laser irradiation inhibits the growth of *S. aureus* at all wavelengths and fluences higher than 12 J/cm^2^. However, for *P. aeruginosa*, photobiomodulation inhibits growth at all wavelengths only at a fluence of 24 J/cm^2^. *E. coli* has similar growth inhibition at a wavelength of 830 nm at fluences of 3, 6, 12, and 24 J/cm^2^. At wavelengths of 660 and 904 nm, growth inhibition is only observed at fluences of 12 and 18 J/cm^2^, respectively.
[108]	*P. aeruginosa*, *E. coli*, *E. faecalis*, *Staphylococcus epidermidis*, *Streptococcus pyogenes*, *Staphylococcus saprophyticus*, *S. aureus*	Ga-Al-As laser (810 nm), 0.36 W/cm^2^, 13 J/cm^2^ for 36 s, 18 J/cm^2^ for 60 s, 30 J/cm^2^ for 80 s, 0.5 cm^2^ 500 Hz, duty cycle of 50% and voltage of 240 V	Photobiomodulation appears to be an effective treatment for Gram-negative and Gram-positive bacterial strains.
[109]	*S. aureus*	Diode laser (830 nm), 0.03 W, 1, 2, 3, 4, 5, and 16 J/cm^2^, 4–8–12–16–20–64 s, CW Diode laser (660 nm), 0.03 W, 1, 2, 3, 4, 5, and 16 J/cm^2^, 2–4–6–8–10–32 s, CW Diode laser (904 nm), 0.04 W, 1, 2, 3, 4, 5, and 16 J/cm^2^, 3, 6, 9, 12, 15, and 48 s, 9500 Hz, duty cycle of 0.1%	Photobiomodulation at 830 and 904 nm wavelengths reduces the growth of S. aureus. Specifically, the most evident topographical change of the cell structure occurred irradiating at 904 nm at a fluence of 3 J/cm^2^.
[110]	*S. aureus*, *P. aeruginosa*, *E. coli*	Laser (450 nm), 0.07 W, 3–6–12–18–24 J/cm^2^, 43–86–172–257–343 s, 1 cm^2^, CW	Blue laser light is capable of inhibiting bacterial growth at low fluences over time, thus presenting no time-dependent effect.
[111]	*S. mutans*, *Lactobacillus casei*, *Actinomyces naeslundii*	Diode laser (810 nm), 1–1.5 W, 30 s, flexible the optic fiber of 200 µm diameter	Diode lasers exert an antibacterial effect of varying levels against all three cariogenic bacteria.
[112]	*S. mutans*	InGaAsP diode laser (780 nm), 0.04 W, 5–10–20 J/cm^2^, 250–500–1000 s, CW	Photobiomodulation has an inhibitory effect on the microorganisms, and this capacity can be altered according to the interactions between different microbial species.
[113]	*S. aureus*, *E. coli*	Semiconductor lasers (405 nm and 445 nm), power in the range of 0.05–0.50 W, 0.050 W/cm^2^, 0–10800 s, CW	*S. aureus* and *E. coli* are inhibited in growth by a dose-dependent modality. 405 nm is more effective than 445 nm.
[114]	*P. intermedia*, *Prevotella nigrescens*	Light-emitting diode (405 nm), 0.019 W/cm^2^, 0.19–0.57–1.14–5.73 J/cm^2^, 10–30–60–300 s, 0.79 cm^2^	Lethal photosensitivity is demonstrated in two species of *Prevotella* spp. under anaerobic conditions.
[115]	*P. gingivalis*	LED (460 nm), 0.40 W/cm^2^, 1–10–100 J	Photobiomodulation has a bactericidal effect on potential multiple drug-resistant bacteria.
[116]	*P. aeruginosa*	LED (415 nm), 19.5 mW/cm^2^, 14–28–56.1–84.2–109.9 J/cm^2^, from 720 to 5760 s	Blue light therapy might offer an effective and safe alternative to conventional antimicrobial therapy for *P. aeruginosa* burn infections.
[117]	*P. gingivalis*	LED (from 400 to 700 nm), 0.05 W/cm^2^ for 300 s, 0.20 W/cm^2^ for 75 s, 0.40 W/cm^2^ for 38 s, 15 J/cm^2^	*P. gingivalis* growth is specifically suppressed by 405 nm light irradiation, suggesting that visible blue light irradiation is a promising means for eradicating periodontal pathogenic bacteria from periodontal lesions.
[118]	*P. aeruginosa*, *S. aureus*	Ar ion laser (514 nm); SHG Nd:YAG (532 nm); He-Ne laser (633 nm), 0.3–19 mW; 0.015–1.130 J/cm^2^; 350 and 420 s; CW	The laser can affect cell growth. The three wavelengths induce a proliferative effect on *P. aeruginosa* and an inhibitory effect on *S. aureus*.
[119]	*E. coli*	He-Ne laser (632.8 nm); Filament-lamp (631 nm), 4–40 mW; 0.01–10 J/cm^2^; CW	The laser increases cell growth. The wavelengths increase DNA synthesis and proliferation.
[120]	*E. coli*	InGaAsP-InP laser diode (1300 nm), 0.03 W/cm^2^, 0.9–9 J/cm^2^, 30–300 s, CW GaAs semiconductor diode (950 nm), 120 W/m^2^, 2–26–700–1000–5000 Hz and an equivalent pulse duration of 410–31.1–1.15–0.82–0.16 ms, respectively	The laser increases cell growth. 1300 nm laser diode increases the division of *E. coli* with an influence range of 0.9–9 J/cm^2^. 950 nm pulsed irradiation increases or inhibits the division rate of bacteria depending on the pulsing frequency and/or pulse duration.
[121]	*E.* *coli*	He-Ne laser (632.8 nm); semiconductor lasers (1066 and 1286 nm), from 0.03 to 30 W/cm^2^, from 0.05 to 2000 J/cm^2^, from 10^−2^ to 900 s	The laser increase cell growth. Irradiation times produce two maxima effects in the growth stimulation. First at 0.5 J/cm^2^ and then at 60–3000 J/cm^2^.
[122]	*E. coli* (different genotypes)	He-Ne laser (632.8 nm); 15 mW; 0.4–4.0 J/cm^2^; CW	The laser increases cell growth. The growth and protein synthesis of *E. coli* are affected by laser light based on the cell cycle phase and the strain genotype.
[123]	*Aggregatibacter actinomycetemcomitans*, *P. gingivalis*, *P. nigrescens*, *F. nucleatum*	InGaAlP laser (660 nm), 0.035 W, 74.2 J/cm^2^, 2.1 J/point, 60 s	Inhibition of cell growth.

**Table 2:** Sensitivity of oral bacteria to different photobiomodulation therapies. Microorganisms were selected according to Table 1.

Bacteria	Best Therapy Effect (Inhibition of Cell Growth, Death) Reported in the Selected Literature	Reference
*Porphyromonas gingivalis* Gram-negative, anaerobic, black-pigmented	Type of cell culture: medium Plasma-arc (450–490 nm), 1.144 W/cm^2^, 138 J/cm^2^, 1 cm^2^, 120 s, CW LED (450–480 nm), 0.520 W/cm^2^, 62 J/cm^2^, 1 cm^2^, 120, CW Halogen lamps (model 1, 400–500 nm), 0.416 W/cm^2^, 75 J/cm^2^, 1 cm^2^, 180 s Killed bacteria: ~100% Halogen lamps (model 2, 400–500 nm), 0.260 W/cm^2^, 47 J/cm^2^, 1 cm^2^, 180 s Killed bacteria: ~80–90% Type of cell culture: Agar Similar effects described above	[103]
	Type of cell culture: medium Argon laser (488 nm + 514 nm), 0.58 W, 0.15 W/cm^2^, 18 J/cm^2^, 3.5–4 cm^2^, 120 s, CW Killed bacteria: ~25–30%	[104]
	Type of cell culture: medium LED (460 nm), 0.40 W/cm^2^, 100 J Killed bacteria: ~50%	[115]
	Type of cell culture: medium LED 405 nm, 0.05 W/cm^2^ for 300 s, 0.20 W/cm^2^ for 75 s, 0.40 W/cm^2^ for 38 s, 15 J/cm^2^ Inhibition bacteria growth: ~75%	[117]
	Organism: rat InGaAlP laser (660 nm), 0.035 W, 74.2 J/cm^2^, 2.1 J/point, 60 s; 4 applications Killed bacteria: ~50% respect to baseline	[123]
*Prevotella intermedia* Gram-negative, anaerobic, black-pigmented	Type of cell culture: medium Argon laser (488 nm + 514 nm), 0.58 W, 0.15 W/cm^2^, 120 J/cm^2^, 3.5–4 cm^2^, 800 s, CW Killed bacteria (strain 15033): ~100% Killed bacteria (strain 49046): ~0%	[104]
	Type of cell culture = Agar Light-emitting diode (405 nm), 0.019 W/cm^2^, 1.14 J/cm^2^, 60 s, 0.79 cm^2^ Killed bacteria: ~40%	[114]
*Prevotella nigrescens* Gram-negative, anaerobic, black-pigmented	Type of cell culture: Agar Light-emitting diode (405 nm), 0.019 W/cm^2^, 5.7 J/cm^2^, 300 s, 0.79 cm^2^ Killed bacteria: ~13%	[114]
	Organism: rat InGaAlP laser (660 nm), 0.035 W, 74.2 J/cm^2^, 2.1 J/point, 60 s; 4 applications Surviving bacteria: ~0% respect to baseline	[123]
*Fusobacteriurn nucleatun* Gram-negative, anaerobic	Type of cell culture: medium Halogen lamps (model 2, 400–500 nm), 0.260 W/cm^2^, 39 J/cm^2^, 1 cm^2^, 150 s Killed bacteria: ~90–100% LED (450–480 nm), 0.520 W/cm^2^, 93 J/cm^2^, 1 cm^2^, 120, CW Killed bacteria: ~50% Halogen lamps (model 2, 400–500 nm), 0.416 W/cm^2^, 62 J/cm^2^, 1 cm^2^, 150 s Killed bacteria: ~100% Plasma-arc (450–490 nm), 1.144 W/cm^2^, 207 J/cm^2^, 1 cm^2^, 180 s, CW Killed bacteria: ~40% Type of cell culture: Agar Killed bacteria: they were almost totally killed through 150 s of irradiation under all experimental conditions	[103]
	Organism: rat InGaAlP laser (660 nm), 0.035 W, 74.2 J/cm^2^, 2.1 J/point, 60 s; 4 applications Killed bacteria: ~40–45% with respect to baseline	[123]
*Escherichia coli* Gram-negative, facultative anaerobic	Type of cell culture: medium Argon-ion pumped tunable dye laser (630 nm); 0.015 W/cm^2^, 1 J/cm^2^, 66 s, CW Inhibition bacteria growth: ~25%	[105]
	Type of cell culture: medium Diode laser (830 nm), 0.03 W, 24 J/cm^2^, 800 s, CW Inhibition bacteria growth: ~50%	[107]
	Type of cell culture: medium Ga-Al-As laser 810 nm, 0.36 W/cm^2^, 13 J/cm^2^ for 36 s, 30 J/cm^2^ for 80 s, 0.5 cm^2^ 500 Hz, duty Cycle of 50% and voltage of 240 V Large effect on inhibition bacteria growth	[108]
	Type of cell culture: medium Laser (450 nm), 0.07 W, 6 and 12 J/cm^2^, 86 and 172 s, 1 cm^2^, CW Killed bacteria: ~40%	[110]
	Type of cell culture: Agar Semiconductor lasers (405 nm), power in the range of 0.05–0.50 W, 0.050 W/cm^2^, 180 J/cm^2^, 3600 s, CW Killed bacteria: ~100%	[113]
*Pseudomonas aeruginosa* Gram-negative, aerobic	Type of cell culture: medium Argon-ion pumped tunable dye laser (630); 0.015 W/cm^2^, 1 J/cm^2^, 66 s, CW Diode lasers (810), 0.015 W/cm^2^, 5 J/cm^2^, 330 s, CW Inhibition bacteria growth: ~25%	[105]
	Type of cell culture: medium Diode lasers (810), 0.015 W/cm^2^, 5 J/cm^2^, 330 s, CW Inhibition bacteria growth: ~25%	[106]
	Type of cell culture: medium Diode laser (660 or 830 nm), 0.03 W, 24 J/cm^2^, 800 s, CW Diode laser (904 nm), 0.04 W, 24 J/cm^2^, 600 s CW Inhibition bacteria growth: ~50%	[107]
	Type of cell culture: medium Ga-Al-As laser 810 nm, 0.36 W/cm^2^, 13 J/cm^2^ for 36 s, 0.5 cm^2^ 500 Hz, duty cycle of 50% and voltage of 240 V Large effect on inhibition bacteria growth	[108]
	Type of cell culture: medium Laser (450 nm), 0.07 W, 18 and 24 J/cm^2^, 257 and 343 s, 1 cm^2^, CW Killed bacteria: ~60%	[110]
	Type of cell culture: medium LED (415 nm), 0.0195 W/cm^2^, 109.9 J/cm^2^, 5760 s, Killed bacteria: ~100%	[116]
*Staphylococcus aureus* Gram-positive, facultative anaerobic	Type of cell culture: medium Argon-ion pumped tunable dye laser (630 nm); 0.015 W/cm^2^, 5 J/cm^2^, 330 s, CW Diode lasers (810 nm and 905 nm), 0.015 W/cm^2^, 5 J/cm^2^, 330 s, CW Inhibition bacteria growth: ~10	[105]
	Type of cell culture: medium Diode laser (810 nm), 0.015 W/cm^2^; 1 and 2 J/cm^2^; 66 ans 132 s, 50% duty cycle; 292 Hz Inhibition bacteria growth: ~10%	[106]
	Type of cell culture: medium Diode laser (660 nm), 0.03 W, 24 J/cm^2^, 800 s, CW Inhibition bacteria growth: ~78%	[107]
	Type of cell culture: medium Diode laser (904 nm), 0.04 W, 3 J/cm^2^, 9 s, 9500 Hz, duty cycle of 0.1% Killed bacteria: ~80%	[109]
	Type of cell culture: medium Laser (450 nm), 0.07 W, 6, 12, 18, and 24 J/cm^2^, 86–172–257 and 343 s, 1 cm^2^, CW Killed bacteria: ~60%	[110]
	Type of cell culture: Agar Semiconductor lasers (405 nm), power in the range of 0.05–0.50 W, 0.050 W/cm^2^, 180 J/cm^2^, 3600 s, CW Killed bacteria: ~92%	[113]
	Type of cell culture: medium He-Ne laser (633 nm), 0.019 W; 1.130 J/cm^2^; 420 s; CW Surviving bacteria: ~33%	[118]
*Streptococcus mutans* Gram-positive, facultative anaerobic	Type of cell culture: medium Plasma-arc (450–490 nm), 1.144 W/cm^2^, 159 J/cm^2^, 1 cm^2^, 138 s, CW Killed bacteria: inhibition of cell growth (not specified)	[103]
	Type of cell culture: medium Diode laser (810 nm), 1.5 W, 30 s, flexible optic fiber of 200 µm diameter Killed bacteria: ~70%	[111]
	Type of cell culture: medium InGaAsP diode laser (780 nm), 0.04 W, 0.02 W/cm^2^, 20 J/cm^2^, 1000 s, CW Biofilm reduction: ~90%	[112]
*Streptococcus pyogenes* Gram-positive, facultative anaerobic	Type of cell culture: medium Ga-Al-As laser (810 nm), 0.36 W/cm^2^, 18 J/cm^2^ for 60 s, 30 J/cm^2^ for 80 s, 0.5 cm^2^, 500 Hz, duty cycle of 50% and voltage of 240 V Large effect on inhibition bacteria growth	[108]
*Enterococcus faecalis* Gram-positive, facultative anaerobic	Type of cell culture: medium Plasma-arc (450–490 nm), 1.144 W/cm^2^, 212 J/cm^2^, 1 cm^2^, 184 s, CW Surviving bacteria: inhibition (not specified)	[103]
	Type of cell culture: medium Ga-Al-As laser (810 nm), 0.36 W/cm^2^, 13 J/cm^2^ for 36 s, 30 J/cm^2^ for 80 s, 0.5 cm^2^, 500 Hz, duty cycle of 50% and voltage of 240 V Large effect on inhibition bacteria growth	[108]
*Staphylococcus epidermidis* Gram-positive, facultative anaerobic	Type of cell culture: medium Ga-Al-As laser 810 nm, 0.36 W/cm^2^, 18 J/cm^2^ for 60 s, 30 J/cm^2^ for 80 s, 0.5 cm^2^ 500 Hz, duty cycle of 50% and voltage of 240 V Large effect on inhibition bacteria growth	[108]
*Staphylococcus saprophyticus* Gram-positive, facultative anaerobic	Type of cell culture: medium Ga-Al-As laser 810 nm, 0.36 W/cm^2^, 18 J/cm^2^ for 60 s, 30 J/cm^2^ for 80 s, 0.5 cm^2^ 500 Hz, duty cycle of 50% and voltage of 240 V Large effect on inhibition bacteria growth	[108]
*Lactobacillus casei* Gram-positive, facultative anaerobic	Type of cell culture: medium Diode laser (810 nm), 1.5 W, 30 s, flexible optic fiber of 200 µm diameter Killed bacteria: ~50%	[111]
*Actinomyces naeslundii* Gram-positive, anaerobic	Type of cell culture: medium Diode laser (810 nm), 1.5 W, 30 s, flexible optic fiber of 200 µm diameter Killed bacteria: ~38%	[111]

## Data Availability

Data sharing is not applicable to this article as no new data were created or analyzed in this study.

## References

[1] Witzany G. (2016). Crucial steps to life: From chemical reactions to code using agents. Biosystems.

[2] Marijuán P.C., Navarro J. (2021). From Molecular Recognition to the “Vehicles” of Evolutionary Complexity: An Informational Approach. Int. J. Mol. Sci..

[3] Trevors J.T. (2012). Origin of life: Hypothesized roles of high-energy electrical discharges, infrared radiation, thermosynthesis and pre-photosynthesis. Theory Biosci..

[4] Barge L.M. (2018). Considering planetary environments in origin of life studies. Nat. Commun..

[5] Oparin A.I. (1968). Genesis and Evolutionary Development of Life.

[6] Urey H.C. (1952). The Planets: Their Origin and Development.

[7] Rubey W.W. (1951). Geological history of sea water: An attempt to state the problem. Bull. Geol. Soc. Am..

[8] Holland H.D., Engel A.E., James H.L., Leonard B.F. (1962). Model for the evolution of the Earth’s atmosphere. Petrologic Studies: A Volume to Honour A.F. Buddington.

[9] Luisi P.L. (2006). The Emergence of Life.

[10] Dalai P., Sahai N. (2019). Mineral-Lipid Interactions in the Origins of Life. Trends Biochem. Sci..

[11] Baur M.E. (1978). Thermodynamics of heterogeneous iron·carbon systems. Implications for the terrestrial primitive reducing atmosphere. Chem. Ceol..

[12] Lu A., Li Y., Ding H. (2019). Photoelectric conversion on Earth’s surface via widespread Fe- and Mn-mineral coatings. Proc. Natl. Acad. Sci. USA.

[13] Fox S.W., Dose K. (1972). Molecular Evolution and the Origins of Life.

[14] Kritsky M.S., Telegina T.A., Buglak A.A. (2014). Modeling of abiotic ATP synthesis in the context of problems of early biosphere evolution. Geochem. Int..

[15] Kolesnikov M.P., Kritsky M.S. (2001). Study of chemical structure and of photochemical activity of biogenic flavin pigment. J. Evol. Biochem. Physiol..

[16] Losi A. (2007). Flavinbased bluelight photosensors: A photobiophysics update. Photochem. Photobiol..

[17] Bahn P.R., Fox S.W. (1981). Models for protocellular photophosphorylation. BioSystems.

[18] Kritsky M.S., Kolesnikov M.P., Telegina T.A. (2007). Modeling of abiogenic synthesis of ATP. Dokl. Biochem. Biophys..

[19] Krasnovsky A.A., Umrikhina A.V. (1964). On abiogenic formation of porphin and its role in the process of photochemical electron transport. Dokl. ANSSSR.

[20] Szutka A. (1964). porphine-like substances: Probable synthesis during chemical evolution. Nature.

[21] Olson J.M., Pierson B.K. (1987). Origin and evolution of photosynthetic reaction centers. Orig. Life Evol. Biosph..

[22] Lozovaya G.I., Masinovsky Z., Sivash A.A. (1990). Protoporphyrin ix as a possible ancient photosensitizer: Spectral and photochemical studies. Orig. Life Evol. Biosph..

[23] Kolesnikov M.P., Telegina T.A., Lyudnikova T.A., Kritsky M.S. (2008). Abiogenic photophosphorylation of ADP to ATP sensitized by flavoproteinoid microspheres. Orig. Life Evol. Biosph..

[24] Nelson K.E., Levy M., Miller S.L. (2000). Peptide nucleic acids rather than RNA may have been the first genetic molecule. Proc. Natl. Acad. Sci. USA.

[25] Tamulis A., Ruksenas O. (2008). Quantum mechanical interpretation of the origin of life. Science in the Faculty of Natural Sciences of Vilnius University.

[26] Dzieciol A.J., Mann S. (2012). Designs for life: Protocell models in the laboratory. Chem. Soc. Rev..

[27] Soo R.M., Hemp J., Parks D.H., Fischer W.W., Hugenholtz P. (2017). On the origins of oxygenic photosynthesis and aerobic respiration in Cyanobacteria. Science.

[28] Sessions A.L., Doughty D.M., Welander P.V., Summons R.E., Newman D.K. (2009). The continuing puzzle of the great oxidation event. Curr. Biol..

[29] Schäfer G., Engelhard M., Müller V. (1999). Bioenergetics of the Archaea. Microbiol. Mol. Biol. Rev..

[30] Esposti M.D. (2020). On the evolution of cytochrome oxidases consuming oxygen. Biochim. Biophys. Acta Bioenerg..

[31] Schäfer G., Purschke W., Schmidt C.L. (1996). On the origin of respiration: Electron transport proteins from archaea to man. FEMS Microbiol. Rev..

[32] Castresana J., Saraste M. (1995). Evolution of energetic metabolism: The respiration-early hypothesis. Trends Biochem. Sci..

[33] Woese C.R., Kandler O., Wheelis M.L. (1990). Towards a natural system of organisms, proposal for the domains archaea, bacteria and eucarya. Proc. Natl. Acad. Sci. USA.

[34] Gogarten J.P., Iaiz L. (1992). Evolution of proton pumping ATPases: Rooting the tree of life. Photosynth. Res..

[35] Kracke F., Vassilev I., Krömer J.O. (2015). Microbial electron transport and energy conservation—The foundation for optimizing bioelectrochemical systems. Front. Microbiol..

[36] Sousa F.L., Alves R.J., Ribeiro M.A., Pereira-Leal J.B., Teixeira M., Pereira M.M. (2012). The superfamily of heme-copper oxygen reductases: Types and evolutionary considerations. Biochim. Biophys. Acta.

[37] Hernandez M., Newman D. (2001). Extracellular electron transfer. Cell. Mol. Life Sci..

[38] John P., Whatley F. (1975). *Paracoccus denitrificans* and the evolutionary origin of the mitochondrion. Nature.

[39] Van Spanning R.J., de Boer A.P., Reijnders W.N., De Gier J.W., Delorme C.O., Stouthamer A.H., Westerhoff H.V., Harms N., van der Oost J. (1995). Regulation of oxidative phosphorylation: The flexible respiratory network of *Paracoccus denitrificans*. J. Bioenerg. Biomembr..

[40] Hino T., Matsumoto Y., Nagano S., Sugimoto H., Fukumori Y., Murata T., Iwata S., Shiro Y. (2010). Structural basis of biological N_2_O generation by bacterial nitric oxide reductase. Science.

[41] Moodie A.D., Ingledew W.J. (1990). Microbial anaerobic respiration. Adv. Microb. Physiol..

[42] O’Malley M.A. (2015). Endosymbiosis and its implications for evolutionary theory. Proc. Natl. Acad. Sci. USA.

[43] Martin W.F., Garg S., Zimorski V. (2015). Endosymbiotic theories for eukaryote origin. Philos. Trans. R. Soc. Lond. B Biol. Sci..

[44] Zimorski V., Ku C., Martin W.F., Gould S.B. (2014). Endosymbiotic theory for organelle origins. Curr. Opin. Microbiol..

[45] Koonin E.V. (2010). The origin and early evolution of eukaryotes in the light of phylogenomics. Genome Biol..

[46] Berry S. (2003). Endosymbiosis and the design of eukaryotic electron transport. Biochim. Biophys. Acta.

[47] Archibald J.M. (2015). Endosymbiosis and Eukaryotic Cell Evolution. Curr. Biol..

[48] Bewley M.C., Marohnic C.C., Barber M.J. (2001). The structure and biochemistry of NADH-dependent cytochrome b5 reductase are now consistent. Biochemistry.

[49] Niklas K.J. (1997). The Evolutionary Biology of Plant.

[50] Anders J.J., Lanzafame R.J., Arany P.R. (2015). Low-level light/laser therapy versus photobiomodulation therapy. Photomed. Laser Surg..

[51] Albini A. (2016). Some remarks on the first law of photochemistry. Photochem. Photobiol. Sci..

[52] Pastore D., Greco M., Passarella S. (2000). Specific helium-neon laser sensitivity of the purified cytochrome c oxidase. Int. J. Radiat. Biol..

[53] Karu T.I. (2010). Multiple roles of cytochrome c oxidase in mammalian cells under action of red and IR-A radiation. IUBMB Life.

[54] Amaroli A., Ravera S., Parker S., Panfoli I., Benedicenti A., Benedicenti S. (2016). An 808-nm Diode Laser with a Flat-Top Handpiece Positively Photobiomodulates Mitochondria Activities. Photomed. Laser Surg..

[55] Amaroli A., Pasquale C., Zekiy A., Utyuzh A., Benedicenti S., Signore A., Ravera S. (2021). Photobiomodulation and oxidative stress: 980 nm diode-laser light regulates mitochondria activity and reactive oxygen species production. Oxid. Med. Cell. Longev..

[56] Ravera S., Ferrando S., Agas D., De Angelis N., Raffetto M., Sabbieti M.G., Signore A., Benedicenti S., Amaroli A. (2019). 1064 nm Nd:YAG laser light affects transmembrane mitochondria respiratory chain complexes. J. Biophotonics.

[57] Swartz T.E., Corchnoy S.B., Christie J.M., Lewis J.W., Szundi I., Briggs W.R., Bogomolni R.A. (2001). The photocycle of a flavin-binding domain of the blue light photoreceptor phototropin. J. Biol. Chem..

[58] Buravlev E.A., Zhidkova T.V., Vladimirov Y.A., Osipov A.N. (2014). Effects of low-level laser therapy on mitochondrial respiration and nitrosyl complex content. Lasers Med. Sci..

[59] Koren K., Borisov S.M., Saf R., Klimant I. (2011). Strongly Phosphorescent Iridium(III) Porphyrins—New Oxygen Indicators with Tuneable Photophysical Properties and Functionalities. Eur. J. Inorg. Chem..

[60] Werck-Reichhart D., Feyereisen R. (2000). Cytochromes P450: A success story. Genome Biol..

[61] Colombo E., Signore A., Aicardi S., Zekiy A., Utyuzh A., Benedicenti S., Amaroli A. (2021). Experimental and Clinical Applications of Red and Near-Infrared Photobiomodulation on Endothelial Dysfunction: A Review. Biomedicines.

[62] Wang Y., Huang Y.-Y., Wang Y., Lyu P., Hamblin M.R. (2017). Photobiomodulation of human adipose-derived stem cells using 810 nm and 980 nm lasers operates via different mechanisms of action. Biochim. Biophys. Acta Gen. Subj..

[63] Jansen K., Wu M., van der Steen A.F. (2013). Photoacoustic imaging of human coronary atherosclerosis in two spectral bands. Photoacoustics.

[64] Castellano-Pellicena I., Uzunbajakava N.E., Mignon C., Raafs B., Botchkarev V.A., Thornton M.J. (2019). Does blue light restore human epidermal barrier function via activation of Opsin during cutaneous wound healing?. Lasers Surg. Med..

[65] Verbon E.H., Post J.A., Boonstra J. (2012). The influence of reactive oxygen species on cell cycle progression in mammalian cells. Gene.

[66] Whitaker M., Patel R. (1990). Calcium and cell cycle control. Development.

[67] Villalobo A. (2006). Nitric oxide and cell proliferation. FEBS J..

[68] Ravera S., Colombo E., Pasquale C., Benedicenti S., Solimei L., Signore A., Amaroli A. (2021). Mitochondrial Bioenergetic, Photobiomodulation and Trigeminal Branches Nerve Damage, What’s the Connection? A Review. Int. J. Mol. Sci..

[69] Amaroli A., Ferrando S., Benedicenti S. (2019). Photobiomodulation Affects Key Cellular Pathways of all Life-Forms: Considerations on Old and New Laser Light Targets and the Calcium Issue. Photochem. Photobiol..

[70] Amaroli A., Colombo E., Zekiy A., Aicardi S., Benedicenti S., De Angelis N. (2020). Interaction between Laser Light and Osteoblasts: Photobiomodulation as a Trend in the Management of Socket Bone Preservation—A Review. Biology.

[71] Agas D., Hanna R., Benedicenti S., De Angelis N., Sabbieti M.G., Amaroli A. (2021). Photobiomodulation by Near-Infrared 980-nm Wavelengths Regulates Pre-Osteoblast Proliferation and Viability through the PI3K/Akt/Bcl-2 Pathway. Int. J. Mol. Sci..

[72] Pasquale C., Utyuzh A., Mikhailova M.V., Colombo E., Amaroli A. (2021). Recovery from Idiopathic Facial Paralysis (Bell’s Palsy) Using Photobiomodulation in Patients Non-Responsive to Standard Treatment: A Case Series Study. Photonics.

[73] Cassano P., Petrie S.R., Hamblin M.R., Henderson T.A., Iosifescu D.V. (2016). Review of transcranial photobiomodulation for major depressive disorder: Targeting brain metabolism, inflammation, oxidative stress, and neurogenesis. Neurophotonics.

[74] Hanna R., Dalvi S., Benedicenti S., Amaroli A., Sălăgean T., Pop I.D., Todea D., Bordea I.R. (2020). Photobiomodulation Therapy in Oral Mucositis and Potentially Malignant Oral Lesions: A Therapy Towards the Future. Cancers.

[75] Zadik Y., Arany P.R., Fregnani E.R., Bossi P., Antunes H.S., Bensadoun R.J., Gueiros L.A., Majorana A., Nair R.G., Ranna V. (2019). Mucositis Study Group of the Multinational Association of Supportive Care in Cancer/International Society of Oral Oncology (MASCC/ISOO). Systematic review of photobiomodulation for the management of oral mucositis in cancer patients and clinical practice guidelines. Support. Care Cancer.

[76] Ravera S., Bertola N., Pasquale C., Bruno S., Benedicenti S., Ferrando S., Zekiy A., Arany P., Amaroli A. (2021). 808-nm Photobiomodulation Affects the Viability of a Head and Neck Squamous Carcinoma Cellular Model, Acting on Energy Metabolism and Oxidative Stress Production. Biomedicines.

[77] De Pauli Paglioni M., Araújo A.L.D., Arboleda L.P.A., Palmier N.R., Fonsêca J.M., Gomes-Silva W., Madrid-Troconis C.C., Silveira F.M., Martins M.D., Faria K.M. (2019). Tumor safety and side effects of photobiomodulation therapy used for prevention and management of cancer treatment toxicities. A systematic review. Oral Oncol..

[78] Bensadoun R.J., Epstein J.B., Nair R.G., Barasch A., Raber-Durlacher J.E., Migliorati C., Genot-Klastersky M.T., Treister N., Arany P., Lodewijckx J. (2020). Safety and efficacy of photobiomodulation therapy in oncology: A systematic review. Cancer Med..

[79] Jensen S.B., Mouridsen H.T., Bergmann O.J., Reibel J., Breunner N., Nauntofte B. (2008). Oral mucosal lesions, microbial changes, and taste disturbances induced by adjuvant chemotherapy in breast cancer patients. Oral Surg. Oral Med. Oral Pathol. Oral Radiol. Endod..

[80] Liebert A., Bicknell B., Johnstone D.M., Gordon L.C., Kiat H., Hamblin M.R. (2019). “Photobiomics”: Can Light, Including Photobiomodulation, Alter the Microbiome?. Photobiomodul. Photomed. Laser Surg..

[81] Pflughoeft K.J., Versalovic J. (2012). Human microbiome in health and disease. Annu. Rev. Pathol..

[82] Gilbert J.A., Blaser M.J., Caporaso J.G., Jansson J.K., Lynch S.V., Knight R. (2018). Current understanding of the human microbiome. Nat. Med..

[83] Hord N.G. (2008). Eukaryotic-microbiota cross talk: Potential mechanisms for health benefits of prebiotics and probiotics. Annu. Rev. Nutr..

[84] Cho I., Blaser M. (2012). The human microbiome: At the interface of health and disease. Nat. Rev. Genet..

[85] Di Spirito F., La Rocca M., De Bernardo M., Rosa N., Sbordone C., Sbordone L. (2021). Possible Association of Periodontal Disease and Macular Degeneration: A Case-Control Study. Dent. J..

[86] Di Spirito F., Toti P., Pilone V., Carinci F., Lauritano D., Sbordone L. (2020). The Association between Periodontitis and Human Colorectal Cancer: Genetic and Pathogenic Linkage. Life.

[87] Deo P.N., Deshmukh R. (2019). Oral microbiome: Unveiling the fundamentals. J. Oral Maxillofac. Pathol..

[88] Wade W.G. (2013). The oral microbiome in health and disease. Pharmacol. Res..

[89] Sharma N., Bhatia S., Sodhi A.S., Batra N. (2018). Oral microbiome and health. AIMS Microbiol..

[90] Yamashita Y., Takeshita T. (2017). The oral microbiome and human health. J. Oral Sci..

[91] Irfan M., Delgado R.Z.R., Frias-Lopez J. (2020). The Oral Microbiome and Cancer. Front. Immunol..

[92] Barone A., Chatelain S., Derchi G., Di Spirito F., Martuscelli R., Porzio M., Sbordone L. (2020). Antibiotic’s effectiveness after erupted tooth extractions: A retrospective study. Oral Dis..

[93] Stewart P.S., Costerton J.W. (2001). Antibiotic resistance of bacteria in biofilms. Lancet.

[94] Bunce J., Hellyer P. (2018). Antibiotic resistance and antibiotic prescribing by dentists in England 2007–2016. Br. Dent. J..

[95] Tong Y., Lighthart B. (1997). Solar radiation is shown to select for pigmented bacteria in the ambient outdoor atmosphere. Photochem. Photobiol..

[96] Trushin M.V. (2003). Studies on distant regulation of bacterial growth and light emission. Microbiology.

[97] Lubart R., Lipovski A., Nitzan Y., Friedmann H. (2011). A possible mechanism for the bactericidal effect of visible light. Laser Ther..

[98] Bordea I.R., Hanna R., Chiniforush N., Grădinaru E., Câmpian R.S., Sîrbu A., Amaroli A., Benedicenti S. (2020). Evaluation of the outcome of various laser therapy applications in root canal disinfection: A systematic review. Photodiagn. Photodyn. Ther..

[99] Bicknell B., Liebert A., Johnstone D., Kiat H. (2019). Photobiomodulation of the microbiome: Implications for metabolic and inflammatory diseases. Lasers Med. Sci..

[100] Thomé Lima A.M.C., da Silva Sergio L.P., da Silva Neto Trajano L.A., de Souza B.P., da Motta Mendes J.P., Cardoso A.F.R., Figueira C.P., Dos Anjos Tavares B., Figueira D.S., Mencalha A.L. (2020). Photobiomodulation by dualwavelength low-power laser effects on infected pressure ulcers. Lasers Med. Sci..

[101] Amaroli A., Ferrando S., Pozzolini M., Gallus L., Parker S., Benedicenti S. (2018). The earthworm *Dendrobaena veneta* (Annelida): A new experimental-organism for photobiomodulation and wound healing. Eur. J. Histochem..

[102] Feuerstein O., Persman N., Weiss E.I. (2004). Phototoxic effect of visible light on *Porphyromonas gingivalis* and *Fusobacterium nucleatum*: An in vitro study. Photochem. Photobiol..

[103] Henry C.A., Judy M., Dyer B., Wagner M., Matthews J.L. (1995). Sensitivity of Porphyromonas and Prevotella species in liquid media to argon laser. Photochem. Photobiol..

[104] Nussbaum E.L., Lilge L., Mazzulli T. (2002). Effects of 630-, 660-, 810-, and 905-nm laser irradiation delivering radiant exposure of 1–50 J/cm^2^ on three species of bacteria in vitro. J. Clin. Laser Med. Surg..

[105] Nussbaum E.L., Lilge L., Mazzulli T. (2002). Effects of 810 nm laser irradiation on in vitro growth of bacteria: Comparison of continuous wave and frequency modulated light. Lasers Surg. Med..

[106] De Sousa N.T., Gomes R.C., Santos M.F., Brandino H.E., Martinez R., de Jesus Guirro R.R. (2016). Red and infrared laser therapy inhibits in vitro growth of major bacterial species that commonly colonize skin ulcers. Lasers Med. Sci..

[107] Dixit S., Ahmad I., Hakami A., Gular K., Tedla J.S., Abohashrh M. (2019). Comparison of Anti-Microbial Effects of Low-Level Laser Irradiation and Microwave Diathermy on Gram-Positive and Gram-Negative Bacteria in an In Vitro Model. Medicina.

[108] De Sousa N.T., Guirro R.R., Santana H.F., Silva C.C. (2012). In vitro analysis of bacterial morphology by atomic force microscopy of low level laser therapy 660, 830 and 904 nm. Photomed. Laser Surg..

[109] De Sousa N.T., Santos M.F., Gomes R.C., Brandino H.E., Martinez R., de Jesus Guirro R.R. (2015). Blue Laser Inhibits Bacterial Growth of *Staphylococcus aureus*, *Escherichia coli*, and *Pseudomonas aeruginosa*. Photomed. Laser Surg..

[110] Vinothkumar T.S., Apathsakayan R., El-Shamy F.M.M., Homeida H.E., Hommedi A.I.M., Safhi M.Y.A., Alsalhi H.A.M. (2020). Antibacterial effect of diode laser on different cariogenic bacteria: An In-vitro study. Niger. J. Clin. Pract..

[111] Basso F.G., Oliveira C.F., Fontana A., Kurachi C., Bagnato V.S., Spolidório D.M., Hebling J., de Souza Costa C.A. (2011). In vitro effect of low-level laser therapy on typical oral microbial biofilms. Braz. Dent. J..

[112] Plavskii V.Y., Mikulich A.V., Tretyakova A.I., Leusenka I.A., Plavskaya L.G., Kazyuchits O.A., Dobysh I.I., Krasnenkova T.P. (2018). Porphyrins and flavins as endogenous acceptors of optical radiation of blue spectral region determining photoinactivation of microbial cells. J. Photochem. Photobiol. B.

[113] Hope C.K., Strother M., Creber H.K., Higham S.M. (2016). Lethal photosensitisation of Prevotellaceae under anaerobic conditions by their endogenous porphyrins. Photodiagn. Photodyn. Ther..

[114] Yoshida A., Sasaki H., Toyama T., Araki M., Fujioka J., Tsukiyama K., Hamada N., Yoshino F. (2017). Antimicrobial effect of blue light using *Porphyromonas gingivalis* pigment. Sci. Rep..

[115] Dai T., Gupta A., Huang Y.Y., Yin R., Murray C.K., Vrahas M.S., Sherwood M.E., Tegos G.P., Hamblin M.R. (2013). Blue light rescues mice from potentially fatal *Pseudomonas aeruginosa* burn infection: Efficacy, safety, and mechanism of action. Antimicrob. Agents Chemother..

[116] Fukui M., Yoshioka M., Satomura K., Nakanishi H., Nagayama M. (2008). Specific-wavelength visible light irradiation inhibits bacterial growth of *Porphyromonas gingivalis*. J. Periodont. Res..

[117] Dadras S., Mohajerani E., Eftekhar F., Hosseini M. (2006). Different photoresponses of *Staphylococcus aureus* and *Pseudomonas aeruginosa* to 514, 532, and 633 nm low level lasers in vitro. Curr. Microbiol..

[118] Karu T.I., Tiphlova O.A., Letokhov V.S., Lobko V.V. (1983). Stimulation of *E. coli* growth by laser and incoherent red light. Il Nuovo Cimento D.

[119] Karu T., Tiphlova O., Samokhina M., Diamantopoulos C., Sarantsev V.P., Shveikin V. (1990). Effects of near-infrared laser and superluminous diode irradiation on *Escherichia coli* division rate. IEEE J. Quantum Eletron..

[120] Karu T., Tiphlova O., Esenaliev R., Letokhov V. (1994). Two different mechanisms of low-intensity laser photobiological effects on *Escherichia coli*. J. Photochem. Photobiol. B.

[121] Bertoloni G., Sacchetto R., Baro E., Ceccherelli F., Jori G. (1993). Biochemical and morphological changes in *Escherichia coli* irradiated by coherent and non-coherent 632.8 nm light. J. Photochem. Photobiol. B.

[122] Theodoro L.H., Longo M., Ervolino E., Duque C., Ferro-Alves M.L., Assem N.Z., Louzada L.M., Garcia V.G. (2016). Effect of low-level laser therapy as an adjuvant in the treatment of periodontitis induced in rats subjected to 5-fluorouracil chemotherapy. J. Periodont. Res..

[123] Leanse L.G., Dos Anjos C., Mushtaq S., Dai T. (2022). Antimicrobial blue light: A ‘Magic Bullet’ for the 21st century and beyond?. Adv. Drug Deliv. Rev..

[124] De Souza da Fonseca A., da Silva Sergio L.P., Mencalha A.L., de Paoli F. (2021). Low-power lasers on bacteria: Stimulation, inhibition, or effectless?. Lasers Med. Sci..

[125] Lushchak V.I. (2001). Oxidative stress and mechanisms of protection against it in bacteria. Biochemistry.

[126] Ezraty B., Gennaris A., Barras F. (2017). Oxidative stress, protein damage and repair in bacteria. Nat. Rev. Microbiol..

[127] Das A., Silaghi-Dumitrescu R., Ljungdahl L.G., Kurtz D.M. (2005). Cytochrome bd oxidase, oxidative stress, and dioxygen tolerance of the strictly anaerobic bacterium *Moorella thermoacetica*. J. Bacteriol..

[128] Verkhratsky A., Parpura V. (2014). Calcium signalling and calcium channels: Evolution and general principles. Eur. J. Pharmacol..

[129] Yang W.Z., Chen J.Y., Yu J.T., Zhou L.W. (2007). Effects of low power laser irradiation on intracellular calcium and histamine release in RBL-2H3 mast cells. Photochem. Photobiol..

[130] Smalley J.W., Silver J., Birss A.J., Withnall R., Titler P.J. (2003). The haem pigment of the oral anaerobes *Prevotella nigrescens* and *Prevotella intermedia* is composed of iron(III) protoporphyrin IX in the monomeric form. Microbiology.

[131] Soukos N.S., Som S., Abernethy A.D., Ruggiero K., Dunham J., Lee C., Doukas A.G., Goodson J.M. (2005). Phototargeting oral black-pigmented bacteria. Antimicrob. Agents Chemother..

[132] Amaroli A., Barbieri R., Signore A., Marchese A., Parker S., De Angelis N., Benedicenti S. (2020). Simultaneous photoablative and photodynamic 810-nm diode laser therapy as an adjunct to non-surgical periodontal treatment: An in-vitro study. Minerva Stomatol..

[133] Naziya N., Rehman M.A., Dixit P.P. (2020). Influence of light wavelengths, light intensity, temperature, and pH on biosynthesis of extracellular and intracellular pigment and biomass of Pseudomonasaeruginosa NR1. J. King Saud Univ. Sci..

[134] Marsh P.D., Do T., Beighton D., Devine D.A. (2016). Influence of saliva on the oral microbiota. Periodontology 2000.

[135] Rusthen S., Kristoffersen A.K., Young A. (2019). Dysbiotic salivary microbiota in dry mouth and primary Sjögren’s syndrome patients. PLoS ONE.

[136] Lynge Pedersen A.M., Belstrøm D. (2019). The role of natural salivary defences in maintaining a healthy oral microbiota. J. Dent..

[137] Sousa A.S., Silva J.F., Pavesi V.C.S. (2020). Photobiomodulation and salivary glands: A systematic review. Lasers Med. Sci..

[138] Li H., Sun T., Liu C., Cao Y., Liu X. (2020). Photobiomodulation (450 nm) alters the infection of periodontitis bacteria via the ROS/MAPK/mTOR signaling pathway. Free Radic. Biol. Med..

[139] Ailioaie L.M., Litscher G. (2021). Probiotics, Photobiomodulation, and Disease Management: Controversies and Challenges. Int. J. Mol. Sci..

[140] De Castro M.S., Miyazawa M., Nogueira E.S.C., Chavasco J.K., Brancaglion G.A., Cerdeira C.D., Nogueira D.A., Ionta M., Hanemann J.A.C., Brigagão M.R.P.L. (2020). Photobiomodulation enhances the Th1 immune response of human monocytes. Lasers Med. Sci..

[141] Agogué H., Joux F., Obernosterer I., Lebaron P. (2005). Resistance of marine bacterioneuston to solar radiation. Appl. Environ. Microbiol..

[142] Fontes M., Ruiz-Vázquez R., Murillo F.J. (1993). Growth phase dependence of the activation of a bacterial gene for carotenoid synthesis by blue light. EMBO J..

